# Ultrasound-Guided Percutaneous Catheter Drainage in Periappendiceal abscess Management: Retrospective Insights

**DOI:** 10.12669/pjms.41.2.10211

**Published:** 2025-02

**Authors:** Ying-Bin Ding, Wei-Na Wang, Xue-Lei Zhan

**Affiliations:** 1 Ying-Bin Ding, Department of Ultrasound Medicine, Harbin Hospital of Traditional Chinese Medicine, Deputy Chief Physician. Harbin 150076, Heilongjiang, China; 2 Wei-Na Wang, Department of Ultrasound Medicine, Harbin Hospital of Traditional Chinese Medicine, Deputy Chief Physician. Harbin 150076, Heilongjiang, China; 3 Xue-Lei Zhan, Department of General Surgery, the First Hospital of Harbin, Chief Physician, Harbin 150000, Heilongjiang, China

**Keywords:** Periappendiceal abscess, Ultrasound-guided, Percutaneous catheter drainage, Appendectomy, Laparoscopy

## Abstract

**Objective:**

To retrospectively evaluate the comparative efficacy and safety of percutaneous catheter drainage (PCD) combined with antibiotic therapy versus emergency laparoscopic surgery (ELS) for treating periappendiceal abscesses.

**Methods:**

A retrospective study was conducted on 90 patients diagnosed with periappendiceal abscesses at Harbin Hospital of Traditional Chinese Medicine between March 2022 and December 2023. The study compared clinical outcomes between two groups: one receiving percutaneous catheter drainage (PCD) with antibiotic therapy and the other undergoing emergency laparoscopic surgery. The comparison encompassed key outcomes such as operative time, gastrointestinal function recovery, length of hospital stays, postoperative complication rate, recurrence rate, and the need for additional interventions.

**Results:**

The PCD group exhibited a significantly shorter operative time (*P*=0.02) and fewer postoperative complications (*P*=0.008) compared to the ELS group. During outpatient follow-up, six patients in the PCD group had a recurrence of appendicitis, which was statistically significant compared to the ELS group.

**Conclusion:**

Ultrasound-guided PCD has been shown to be effective and safe in the management of periappendiceal abscesses in our single-center clinical study. It offers several benefits over ELS, such as reduced operative time and a decreased incidence of postoperative complications. Our results suggest that PCD, in conjunction with antibiotic therapy, is a feasible alternative to surgery, significantly reducing patient discomfort and healthcare resource consumption. However, these findings are specific to our center, and further multicenter studies are required to confirm these results and to establish guidelines for the optimal application of PCD in clinical practice.

## INTRODUCTION

Periappendiceal abscess, a severe complication of acute appendicitis, presents a significant clinical challenge due to its potential to rapidly worsen a patient’s condition.^1^ Historically, open surgical interventions were the standard treatment; however, the introduction of emergency laparoscopic surgery has marked a shift towards minimally invasive approaches.^2^ Despite these advancements, emergency laparoscopic surgery still has drawbacks, particularly for patients with comorbidities that may increase the risk of postoperative complications and prolong recovery times.^3^

The quest for alternative treatment modalities that reduce these risks while maintaining therapeutic efficacy has led to the exploration of ultrasound-guided PCD. This procedure, facilitated by ultrasound, is a minimally invasive intervention that can be conveniently conducted at the patient’s bedside, thereby eliminating the requirement for general anesthesia and the risks it entails. It involves placing a catheter under real-time ultrasound guidance to drain the purulent collection around the appendix. This method has gained popularity in the medical community, with growing evidence suggesting outcomes that are equivalent to or possibly superior to those of emergency laparoscopic surgery. The principal aim of this retrospective investigation is to adjudicate the relative efficacy and safety of PCD in conjunction with antibiotic therapy, juxtaposed against emergency laparoscopic surgery for the management of periappendiceal abscesses.

This study endeavors to augment the corpus of evidence advocating PCD as a meritorious therapeutic alternative by meticulously scrutinizing clinical endpoints, including operative temporality, recuperation of gastrointestinal functionality, duration of hospital confinement, occurrence of postoperative complications, rate of relapse, and the necessity for further medical interventions. The rationale for adopting PCD is its potential to offer a less traumatic treatment option, beneficial for patients with significant comorbidities or those at high risk for surgical complications.^4^ PCD may also serve as a bridge to surgery in cases where immediate surgical intervention is too risky, allowing for patient stabilization before a definitive procedure. The study was conducted in accordance with the ethical standards of the Ethics Committee of Harbin Hospital of Traditional Chinese Medicine, ensuring patient rights and welfare throughout the research process.

A comprehensive review of medical records from March 2020 to December 2023 was conducted, with meticulous data analysis to compare the clinical outcomes of two distinct treatment approaches. The findings of this study are expected to provide significant insights into the effectiveness of PCD for managing periappendiceal abscesses, which could substantially influence clinical practices and treatment protocols. By demonstrating the safety and efficacy of PCD, this research may prompt a significant shift in the treatment paradigm for this condition, supporting a patient-focused approach that emphasises minimally invasive procedures and expedites recovery.

## METHODS

The retrospective study population comprised 90 patients diagnosed with periappendiceal abscess at Harbin Hospital of Traditional Chinese Medicine from March 2022 to December 2023.

### Ethical Approval:

The study was approved by the Ethics Committee of Harbin Traditional Chinese Medicine Hospital (No. 2022001, Date: 6 March 2022).

### Inclusion & Exclusion criteria:

Inclusion criteria included a confirmed diagnosis of periappendiceal abscess and patients aged 16 years or older. Exclusion criteria comprised patients under 16 years old, those with concurrent pregnancy, patients with immunosuppressive conditions, and patients with an American Society of Anesthesiologists (ASA) classification of four and five.

### Study Groups:

Patients were categorized into two groups based on the treatment approach: Group-I included those who underwent ELS (n=48), while Group-II comprised patients who received PCD under ultrasound guidance (n=42). Randomization was executed using a simple method, wherein each patient was assigned a unique identifier. A computer-generated sequence of random numbers was then used to allocate patients to either Group-I or Group-II, ensuring equal probability for each. This process was overseen by a researcher not involved in clinical decision-making to maintain the study’s integrity.

During the ultrasound-guided drainage of a periappendiceal abscess, patients are positioned supine. A coupling agent is applied to the area overlying the appendix, and an ultrasound probe is used to assess the abscess and its proximity to adjacent structures, thus mapping a safe puncture trajectory. The procedure is performed under local anesthesia with careful monitoring to prevent inadvertent damage to abdominal blood vessels and bowels. It involves puncturing the skin, followed by the insertion of a catheter into the abscess cavity under ultrasound guidance, with adjustments made to the needle’s positioning as necessary. After confirming the abscess, a guide wire is introduced to facilitate the placement of a drainage tube, which is then secured and connected to a drainage system. Post-procedure, patients are closely monitored, and the abscess may be irrigated with saline or antibiotic solutions until the effluent is clear.

Healthcare professionals must maintain a high level of vigilance throughout the process to ensure patient safety and treatment efficacy. After completing the two treatment regimens, patients are administered third-generation cephalosporins and other antibiotics as an interventional measure. Antibiotic therapy continues until the results of antimicrobial susceptibility tests are available, at which point the antibiotic regimen is adjusted accordingly. Therapy is discontinued when the patient exhibits a body temperature below 37.5°C for over 24 hours, a white blood cell (WBC) count below 10×10^9^/L, and resolution of clinical symptoms. Once gastrointestinal function is restored, patients are transitioned to a liquid diet. For those with indwelling drainage tubes, removal is considered when there is no fluid output for at least 24 hours, an ultrasound examination shows no significant pelvic fluid accumulation or abscess, and clinical symptoms have resolved.

### Data Collection:

Demographic and clinical characteristics of patients were documented, including gender, age, main symptoms, duration of symptoms prior to hospital admission, heart rate upon admission, body temperature, WBC count, presence of a mass in the right iliac fossa, size of the abscess, and any accompanying chronic diseases. In the emergency surgery group, the surgical method, operation time, postoperative hospital stays, and postoperative complications were assessed. For the PCD group, the recurrence of appendicitis during the outpatient follow-up period was assessed. After successful drainage by the PCD group, patients must meet the following criteria for discharge: a WBC count below 10×10^9^/L, absence of fever, no abdominal pain or tenderness, and the ability to tolerate oral intake.

### Follow-Up Protocol:

Post-discharge patients are initially followed up in the outpatient department monthly for three months, followed by a follow-up every three months for the subsequent nine months. During outpatient visits, a clinical examination is conducted for all patients. Colonoscopy is routinely performed for patients over the age of 40. Appendectomy is offered to patients with recurrent symptoms of appendicitis. Patients who do not attend outpatient visits are also contacted by phone or WeChat to obtain information about their condition. The duration of follow-up extended from the date of the first visit to the most recent outpatient visit, lasting from 7 to 35 months (average 19.7±11.5 months).

### Data management and statistical analysis:

Data were entered into a secure electronic database and validated for accuracy. Statistical analysis was performed using SPSS version 25.0 (IBM, Armonk, NY, United States). Quantitative data were assessed for normality using the Kolmogorov–Smirnov test and direct data visualisation methods. Numerical data were summarised as means and standard deviations or medians and ranges, depending on the normality testing results. Categorical data were summarised as numbers and percentages. Independent t-tests were used to compare quantitative data between the two groups, while categorical data were compared using the Chi-square test. Multivariate logistic regression analysis was conducted to predict recurrence of appendicitis. Odds ratios and 95% confidence intervals were calculated. The Kaplan-Meier curve was used to analyse time to recurrence. All statistical tests were two-sided, and P values less than 0.05 were considered significant.

## RESULTS

This retrospective study included 90 patients’ data (48 in Group-I and 42 in Group-II). The mean age of patients in Group-I was 53.59±18.86 years, and in Group-II was 55.14±21.32 years. No statistically significant differences were observed in clinical characteristics between Group-I and Group-II (Table-I). In Group-I, 17 cases (35.42%) underwent ileocecal resection due to severe inflammation and adhesion in the terminal ileum region, five cases (10.42%) underwent peritoneal drainage, while the remaining 26 cases (54.17%) underwent appendectomy.

**Table-I:** Comparison of clinical characteristics between ELS group and PCD group.

Characteristics	ELS (n=48)	PCD (n=42)	P-value
Gender (M: F)	26:22	26:16	0.71
Mean Age (years)	53.59±18.86	55.14±21.32	0.34
Duration of Symptoms (days)	7.2±2.1	6.9±2.4	0.58
Pain (100%)	48(100%)	42(100%)	-
Nausea and Vomiting	24 (50%)	21 (50%)	1
Mass in Right Iliac Fossa	21 (43.8%)	19 (45.2%)	0.85
Body Temperature(°C)	37.8±0.9	37.7±1.1	0.47
Heart Rate (pulse/min)	88±15	89±16	0.68
WBC Count (/mm³)	15.2 ± 3.1	15.6 ± 3.3	0.31
Size of Abscess (cm)	5.0 ± 1.2	4.8 ± 1.3	0.22
Comorbidities	10 (20.8%)	9 (21.4%)	0.87

Postoperative complications were noted in 13 patients (27.08%), including wound infection in 9 cases, intra-abdominal hemorrhage in three cases, anastomotic leakage in seven cases, and intra-abdominal abscess in eight cases. No fatalities were recorded for the 48 patients. In Group-II, there were no postoperative complications in the immediate postoperative period. Furthermore, in this group, 11 cases (26.20%) experienced recurrence of appendicitis six weeks after discharge and were treated with laparoscopic appendectomy, with no postoperative complications occurring.

The mean operation time for Group-I was 76.43±18.69 minutes, and for Group-II was 23.13±2.06 minutes. Statistical analysis revealed a statistically significant difference in operation time between the two treatment methods (*P*<0.05). Recovery of gastrointestinal function occurred after 27.85±3.74 hours for Group-I and 2.24±0.44 hours for Group-II, with a statistically significant difference between the groups (*P*<0.05). Normalization of WBC counts was achieved after 4.16±1.35 days for Group-I and 5.68±1.42 days for Group-II, with a statistically significant difference between the groups (*P*<0.05).

Additionally, when comparing the hospital stay between the two groups, Group-I exhibited a shorter hospital stay compared to Group-II, with a statistically significant difference (*P*<0.05). Hospitalization costs were significantly higher for Group-I than for Group-II, with a statistically significant difference (*P*<0.05). (Table-II).

**Table-II:** Comparison of outcomes between ELS group and PCD group.

Characteristics	ELS (n=48)	PCD (n=42)	P-value
Type of Surgery	-	-	-
- Appendectomy	26 (54.17%)	-	-
-Ileocecal Resection	17(35.42%)	-	-
- Peritoneal Drainage Only	5 (10.42%)	-	-
Operation Time (minutes)	76.43 ± 18.69	23.13 ± 2.06	<0.001
Postoperative Complications	13 (27.08%)	0 (0%)	0.008
Hospital Stay (days)	5.1±1.4	3.4±0.8	0.04
Gastrointestinal Function Recovery Time (h)	27.85±3.74	2.24±0.44	0.008
Recurrence Rate	0 (0%)	11 (26.20%)	0.008
Need for Additional Interventions	0 (0%)	11 (26.20%)	0.008

## DISCUSSION

The management of complicated appendicitis lacks standardized guidelines. This retrospective, single-centre study critically evaluates the efficacy of ultrasound-guided PCD for treating periappendiceal abscesses, compared to the conventional approach of ELS. The findings underscore the potential advantages of PCD as a minimally invasive treatment, particularly regarding operative time^5^, postoperative complication rates, and hospitalization costs. The data reveal a significantly shorter operative time for PCD compared with ELS^6^, which not only reduces patient discomfort but also suggests a more efficient use of operating room resources.^7^ A significant benefit in settings with limited medical infrastructure.

Moreover, the lower incidence of postoperative complications in the PCD group supports its role as a conservative treatment option. By avoiding major surgery, PCD mitigates the risk of infections, bleeding, and anastomotic leaks, which is particularly beneficial for patients with comorbidities that increase their risk of adverse postoperative outcomes.^8^ While ELS offers the dual benefits of a reduced incidence of interval surgeries and the ability to concurrently assess and treat additional intra-abdominal pathologies.^9^ Notably, PCD was associated with a faster recovery of gastrointestinal function, indicating a less invasive and less physiologically disruptive procedure.^10^ This aligns with modern surgical practices that emphasize enhanced recovery protocols.^11^

However, the study also noted a higher recurrence rate of abscesses in the PCD group during outpatient follow-up, suggesting that while PCD is effective for immediate management, it may not address the underlying cause of appendicitis as definitively as surgery.^12^ Among patients who were initially treated with antibiotics for uncomplicated acute appendicitis, the likelihood of late recurrence within five years was 39.1%.^13^ This highlights the importance of structured surveillance and follow-up protocols for patients treated with PCD to ensure early detection and management of potential recurrences. Comparisons with other national and international studies show that PCD consistently demonstrates benefits as a minimally invasive option for managing periappendiceal abscesses.^14^ Similar studies from various centres have reported shorter operative times and fewer complications associated with PCD compared to surgical intervention.^15^

However, the recurrence rates observed in our study are consistent with findings from other centres, emphasizing that while PCD may offer immediate advantages^16^, it requires thorough follow-up to manage potential recurrences effectively. The decision-making process should be informed by a thorough assessment of the patient’s condition, anticipated clinical outcomes, and the evidence at hand, rather than focusing solely on the surgeon’s expertise in a specific technique. It is through this balanced approach that optimal patient care can be achieved, harnessing both technical skill and therapeutic efficacy. It is important to acknowledge that the single-centre nature of this study limits the generalisability of our findings. Multi-centre and international studies are necessary to validate these results and provide a broader perspective on the efficacy of PCD in diverse clinical settings. The absence of a randomised control and variations in expertise and resources between centres may also influence outcomes, suggesting that further research with larger, multi-centre cohorts is essential to establish standardized guidelines for managing complicated appendicitis.

### Limitations:

It is a retrospective, single-center study, which may introduce selection biases and incomplete data collection. The applicability of our results is therefore limited to similar settings and patient populations. Future multi-institutional randomized controlled trials (RCTs), with sufficient power, strict selection criteria, clear outcomes, and a homogeneous patient cohort, could provide additional evidence to further clarify the treatment of periappendiceal abscesses.

## CONCLUSION

This study’s empirical data supports the effectiveness of ultrasound-guided PCD in the management of periappendiceal abscesses, suggesting it is superior to ELS. PCD is favored for its rapid intervention, reduced postoperative complications, and quicker recovery. The benefits of PCD include reduced invasiveness, decreased procedural trauma, and significant economic advantages, particularly in terms of hospitalization costs.

However, the higher recurrence rate observed in the PCD group is a significant finding that requires further investigation. This suggests that while PCD is effective for immediate abscess management, it may not address the underlying cause of appendicitis as effectively as surgical interventions. This underscores the need for rigorous postoperative monitoring and a comprehensive follow-up protocol to promptly identify and manage any potential recurrences. The implications of our findings are clear: PCD should be considered as the primary treatment for periappendiceal abscesses, especially when rapid intervention and resource optimization are critical.

Our study contributes to the medical literature by providing a detailed analysis of the clinical outcomes of PCD compared to ELS, offering new insights into the advantages and disadvantages of each approach. The clinical relevance of our findings lies in their potential to guide treatment protocols and enhance patient care by advocating for minimally invasive procedures that prioritize patient comfort and expedite recovery.

### Authors contributions:

**YBD:** Methodology, Investigation, Formal analysis, Writing-Original Draft

**WNW:** Conceptualization, Methodology, Visualization, Project administration, Investigation, Formal analysis

**XLZ:** Supervision, Writing, Review & Editing of the manuscript.

All authors have approved the final version and are accountable le for the integrity of the study.
